# Co-development of a genetic care pathway for ALS: real-world perspectives from the North of England

**DOI:** 10.1186/s13023-026-04417-z

**Published:** 2026-06-17

**Authors:** Clementine Wood, Georgia Brown, Kirsten Chalk, Samantha Holden Smith, Hayley Williams, Greg Broadhurst, John Ealing, Hisham Hamdalla, Catherine Breen, Rhona Macleod, Amina Chaouch

**Affiliations:** 1 Warrington Hospital. Lovely Ln, Warrington, UK; 2 Manchester Centre for Genomic Medicine, St Mary’s Hospital, Manchester University NHS Trust, Manchester, UK; 3 Manchester Centre for Clinical Neurosciences, Salford Royal NHS Foundation Hospital, Northern Care Alliance, Manchester, UK; 4 Motor Neurone Disease Association, Frances Crick House, Moulton Park, Northampton, UK; 5 Division of Evolution, Infection and Genomics, School of Biological Sciences, University of Manchester, Manchester, UK; 6 Faculty of Biology Medicine and Health, University of Manchester, Manchester, UK

**Keywords:** Motor neuron disease (MND), Amyotrophic lateral sclerosis (ALS), Genomic testing, Care pathway

## Abstract

**Background:**

Amyotrophic lateral sclerosis (ALS) is a rare, progressive neurodegenerative disorder, with a substantial proportion of cases attributed to genetic factors. Recent advances in gene discovery and genomic technologies have transformed ALS care by enabling genomic testing to inform prognosis, assess familial risk, and facilitate access to novel therapies. However, guidance on the delivery of genetic testing and counselling in ALS remains limited, leading to variability in clinical practice. In response, the Manchester Motor Neuron Disease (MND) Care Centre and the Manchester Centre for Genomic Medicine co-developed a structured genetic care pathway for ALS, drawing on real-world data, patient engagement, and multidisciplinary collaboration.

**Results:**

A retrospective evaluation of 326 ALS patients at the Manchester MND Care Centre identified significant variability in genetic testing uptake, counselling practices, and record-keeping. Patient survey and engagement sessions revealed uncertainty regarding key genetic concepts and inconsistent recall of pre- and post-test discussions. Priorities for improvement included clearer communication, standardised discussions, and enhanced support for families following genetic findings. Consequently, the Greater Manchester ALS Genetic Testing Pathway was developed by a multidisciplinary team, incorporating consensus-based steps for patient identification, pre-test conversations, consent, testing, results disclosure, and post-test support. This pathway integrates genetic testing into routine ALS care, clarifies team responsibilities, and establishes a framework for ongoing evaluation using key performance indicators. Patient and staff feedback is used to support continuous improvement.

**Conclusions:**

The co-developed ALS genetic testing pathway provides a scalable model for standardising genomic care in mainstream clinical settings. By establishing clear processes for genetics discussions, consent, and follow-up, the pathway seeks to improve equity, transparency, and person-centred care. Ongoing evaluation and collaboration with patients, clinicians, and genetic services are essential to ensure the pathway remains responsive to scientific advances and evolving patient needs. Wider adoption of structured genetic pathways may facilitate the integration of genomics into care for rare diseases across healthcare systems.

## Background

Amyotrophic lateral sclerosis (ALS) is a progressive neurological disorder characterised by degeneration of motor neurons in the brain and spinal cord [1]. ALS is divided into familial (5–10%) and sporadic (90%) forms based on whether affected relatives are reported in the family history. Although familial cases have a higher risk of inheriting the disease, it is becoming clear that a substantial proportion of sporadic cases (up to 21%) will also harbour disease causing changes in the approximately 40 known ALS genes [2]. It remains unclear whether there may be different clinical implications in the absence of a family history. Recent advancements in the identification of novel ALS genes, the development of next-generation sequencing technologies and the availability of new genetic therapies, have substantially transformed the field of ALS genetics [3].

Identification of an ALS-causing genetic variant can, in some individuals, guide prognosis, inform familial risk, and determine eligibility for gene therapy or clinical trials. These findings may also influence relatives considering predictive testing or reproductive options.

The Manchester Motor Neuron Disease (MND) Care Centre delivers support and care for individuals with ALS in Greater Manchester and surrounding areas. The centre delivers coordinated, accessible, and patient-centred care to optimise quality of life for people with ALS. As one of the largest care centres in the United Kingdom (UK), it supports approximately 240 people living with MND at any given time. Several tailored care pathways address the complex needs of patients, including nutrition and gastrostomy feeding, ventilation, cognition, psychological support, and palliative care. Based on a recent 10-year ALS service evaluation, most individuals living with ALS in Greater Manchester experience social deprivation (Index Multiple Deprivation quintile 5) [4]. In collaboration with key partners, the service endeavours to maintain equity of care across the region to meet the needs of those living with ALS.

The National Genomic Test Directory lists genomic tests commissioned by the National Health Service for rare and inherited diseases, describes each test’s technology, and outlines who can access genetic tests in England [5]. The launch of the National Test Directory allows the Manchester MND Care Centre to request genetic testing for any eligible ALS patient, regardless of age or family history. This enables more individuals and their relatives to access genetic testing, research opportunities, and potentially gene therapy.

There is currently limited guidance on the information clinicians should provide to patients before and after genetic testing for ALS. Previous studies have revealed inconsistencies in testing practices among neurologists [6]. A recent National Neurosciences Advisory Group publication has included ALS within its optimal clinical pathway for genetic testing, but does not provide specific guidance concerning pre-and post-test discussions in mainstream clinics [7]. Given the clinical heterogeneity of ALS, there may be additional complexities in familial cases compared to other neurodegenerative diseases. Therefore, recommendations that account for this complexity are necessary.

This paper describes the collaborative development of a care pathway to guide the delivery of genetic testing for patients with ALS. Key areas for improvement were identified based on a recent service evaluation and a patient engagement session. The pathway utilises the RaERN path methodology, a framework for designing rare disease pathways, to support a robust and equitable approach to genetic testing and related discussions [8].

## Methods

### Phase 1: Mapping of existing patient care pathways and patient stories

This phase comprised the collection and analysis of real-world data on the existing genetic testing process for individuals with an established diagnosis of ALS. Both quantitative and qualitative data were obtained concerning the discussion, delivery, and documentation of genomic testing in MND clinics.

**Fig. 1:**
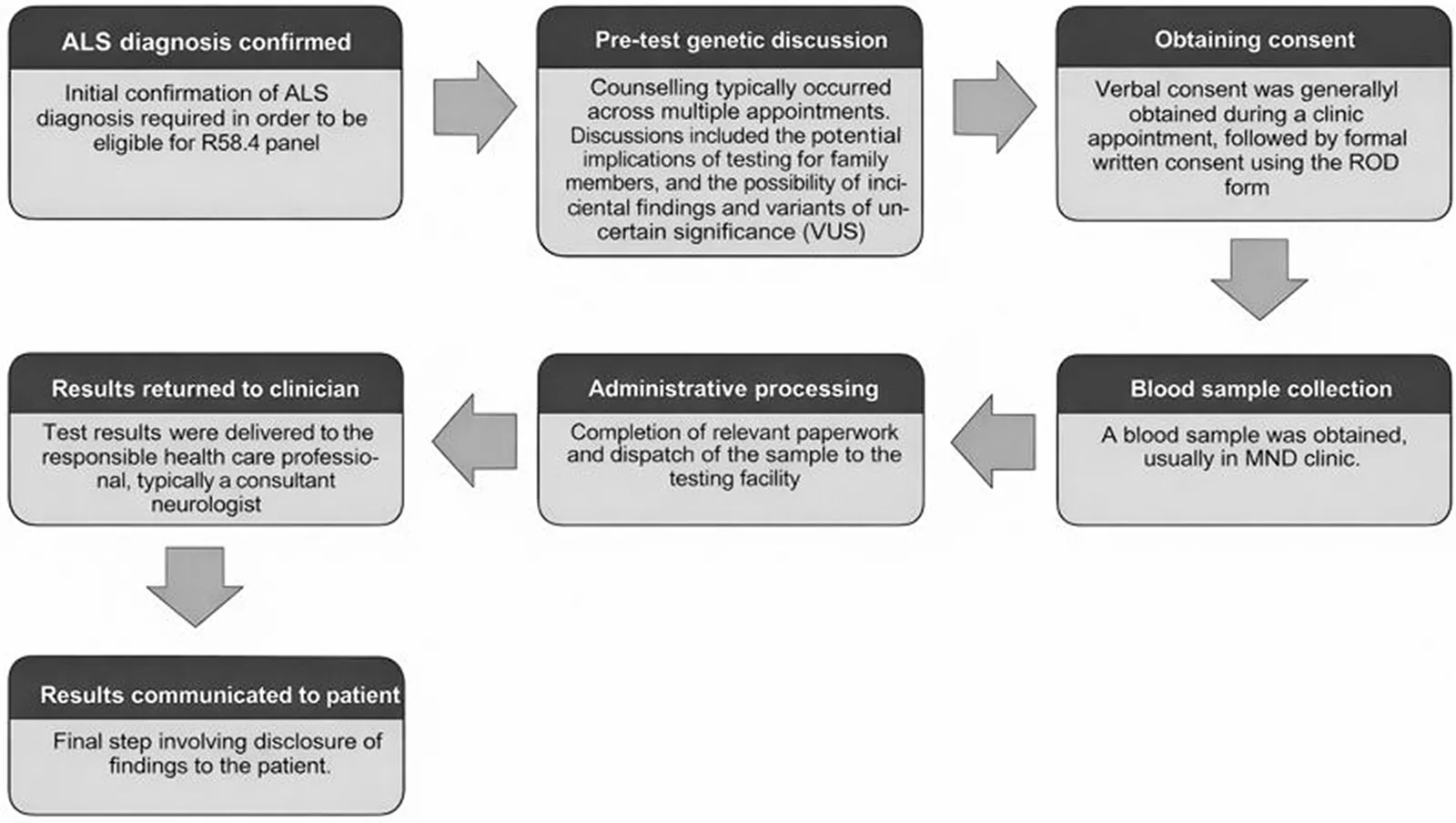
Flowchart summarises the current steps involved in the genetic testing process for patients attending our service

A retrospective service evaluation was conducted with 326 individuals diagnosed with ALS who attended the Manchester MND Care Centre between August 2022 and November 2024. Clinic letters were reviewed to assess uptake of genetic testing, contents of genetics discussions, and the completeness of record keeping (Fig. 1). In this context, pre-test discussion refers to conversations in which patients and relatives are informed about genetic testing, during which they are typically introduced to the Record of Discussion (ROD) form [9].

In the UK, the ROD form is used to consent for whole genome sequencing, including the use and storage of results now and in the future. Pre-test conversations often include information pertaining to ALS genetics that would not be found in the ROD.

### Uptake of genetic testing and results

Of 218 people with documented pre-test conversations, 95 (43.5%) proceeded to have genetic testing. 13 (13.68%) patients (10 *C9orf72*, 3 *CHCHD10*/*TARDBP*/*SOD1*) were found to carry a pathogenic variant. A further 71 (74.7%) patients had no identified variants, while 5 (5.2%) of those tested had results pending or undocumented in the electronic patient record at the time of data collection. The mean turnaround time from referral to release of results was 3.8 months (range 1–18 months). Approximately 46% of counselled patients were offered DNA storage, with 85.1% accepting. All patients with a positive genetic test result were offered referral for genetic counselling while post-test discussions regarding negative results relied on brief written reports that were rarely further discussed in clinic.

### Patient survey: exploring experiences of genetic testing

A patient survey was designed at the Manchester MND Care Centre to evaluate people living with ALS’s understanding, recall, and satisfaction with the genetic testing process. The survey also explored the support provided throughout the testing process.

The survey was structured across several domains:

- Demographic and familial background.
- Timing of genetic discussions.
- Clarity of information.
- Communication of possible outcomes.
- Consent and logistics.
- Follow-up and ongoing support.
- Free-text reflections.

The online survey was distributed to 80 individuals living with ALS and their carers, with 28 responses received. The results indicated considerable variability in experiences with genetic testing. 12 (35%) of respondents did not recall discussions about the implications of a genetic test result for them and their relatives and 9 (33%) did not recall discussing any alternative options to undergoing testing. Discussion of critical topics, such as result uncertainties and implications for family members, was inconsistent. 13 (48%) respondents recalled discussions about DNA storage, while 9 (33%) had a blood test arranged. Among those who underwent testing, timelines for receiving results varied, with 3 (19%) receiving results within 3 months. 15 (68%) individuals reported leaving the clinic without a clear understanding of the next steps.

### Genetics and MND pathway engagement session

A patient engagement session was conducted to present the results of the service evaluation and survey. It was conducted in person with the support of the local MND association branch. 16 individuals attended, including 8 people living with MND and their carers, 3 MND clinicians and the remainder were MND association volunteers (some with family members affected by MND). The session included a presentation outlining progress in developing the care pathway and solicited feedback from participants. The presentation addressed genetic testing outcomes and emphasised the importance of pre-test conversations, particularly in light of interpretive uncertainties and implications for family members.

Respondents expressed concerns regarding their own and their children’s risks. The session identified the need to provide tailored advice and clear guidance. Efforts to expedite urgent results were encouraged, along with managing expectations about waiting times. Respondents requested more information about the storage of their DNA. It was clarified that DNA samples are typically stored long-term and may be accessed by family members, with re-collection required only if sample degradation occurs. Further details were also sought about available options following the identification of a gene change, including research participation and access to gene therapy. Additional requests included increased peer-led support and improved access to educational resources. One suggestion was the development of a ‘genetic hub’ for people living with ALS and their families.

### Phase 2: design of an optimised common patients’ care pathway

Over several months, clinicians specialising in ALS and clinical genetics held a series of meetings to develop a draft pathway for genetic testing in mainstream MND clinics. Findings from the service evaluation and patient survey were reviewed and directly informed the design of the care pathway.

## Results

### Phase 3: consensus on an optimised common patients’ care pathway

The resulting Greater Manchester ALS Genetic Testing Pathway aims to standardise practice, promote equity, and integrate genomic medicine within multidisciplinary ALS care.

#### Step 1 - Identification of eligible patients for testing

Following a confirmed clinical diagnosis of ALS, all patients become eligible for genomic testing in accordance with the National Genomic Testing Directory. Clinicians should record demographic, clinical, and family history information. The timing of discussions should take into account psychological readiness, disease stage, and communication ability.

#### Step 2 - Pre-test conversations

A pre-test discussion is typically conducted by a neurologist or MND nurse. The following topics should be addressed:

- An overview of the genetic basis of ALS and the scope of the genetic test [2].
- The potential outcomes, including identification of a pathogenic or likely pathogenic variant, a variant of uncertain significance, an incidental finding, or no identified variant.
- The implications for the patient and their family, including the availability of reproductive options and the availability of predictive testing.
- The voluntary nature of genetic testing and the option for DNA storage.

Individuals should receive written information from the MND Association and Genomics England, and clinicians are advised to make them aware of the availability of decision aids [10]. If discussions are particularly complex, there is the option of referring to clinical genetics for additional counselling and support before a test is carried out.

#### Step 3 - Requesting a genetic test

Following a discussion, consent is documented on the ROD form. A copy is provided to the patient, and the form is uploaded electronically. The clinician completes the Rare Disease Request Form, using the appropriate test code, at this time, R460 [5]. Both forms, along with the blood sample, are submitted to the regional Genomic Laboratory Hub. Results should be returned to the referring clinician and administrative team after validation. Patients must be informed of the expected turnaround time, which is typically several months in the UK. A clear plan for result disclosure should be established at the time of consent.

#### Step 4 - Results disclosure and post-test conversations

Results are communicated to individuals either in person or by telephone, based on clinical appropriateness and patient preference.

- If a genetic cause is identified (pathogenic or likely pathogenic variant), the clinician explains the nature and implications of the variant, provides a written summary, and issues a “Dear Relative” letter to facilitate referral by a general practitioner for genetic counselling.
- If no genetic cause is identified, the clinician clarifies that the result does not exclude a genetic contribution, explains the potential for future reanalysis, and advises on recontact procedures should variant classification change.

Supporting materials from the MND Association and Genomics England are available to facilitate post-test discussions.

#### Step 5 - Integration of Genetic Testing within the ALS Clinic

**Fig. 2:**
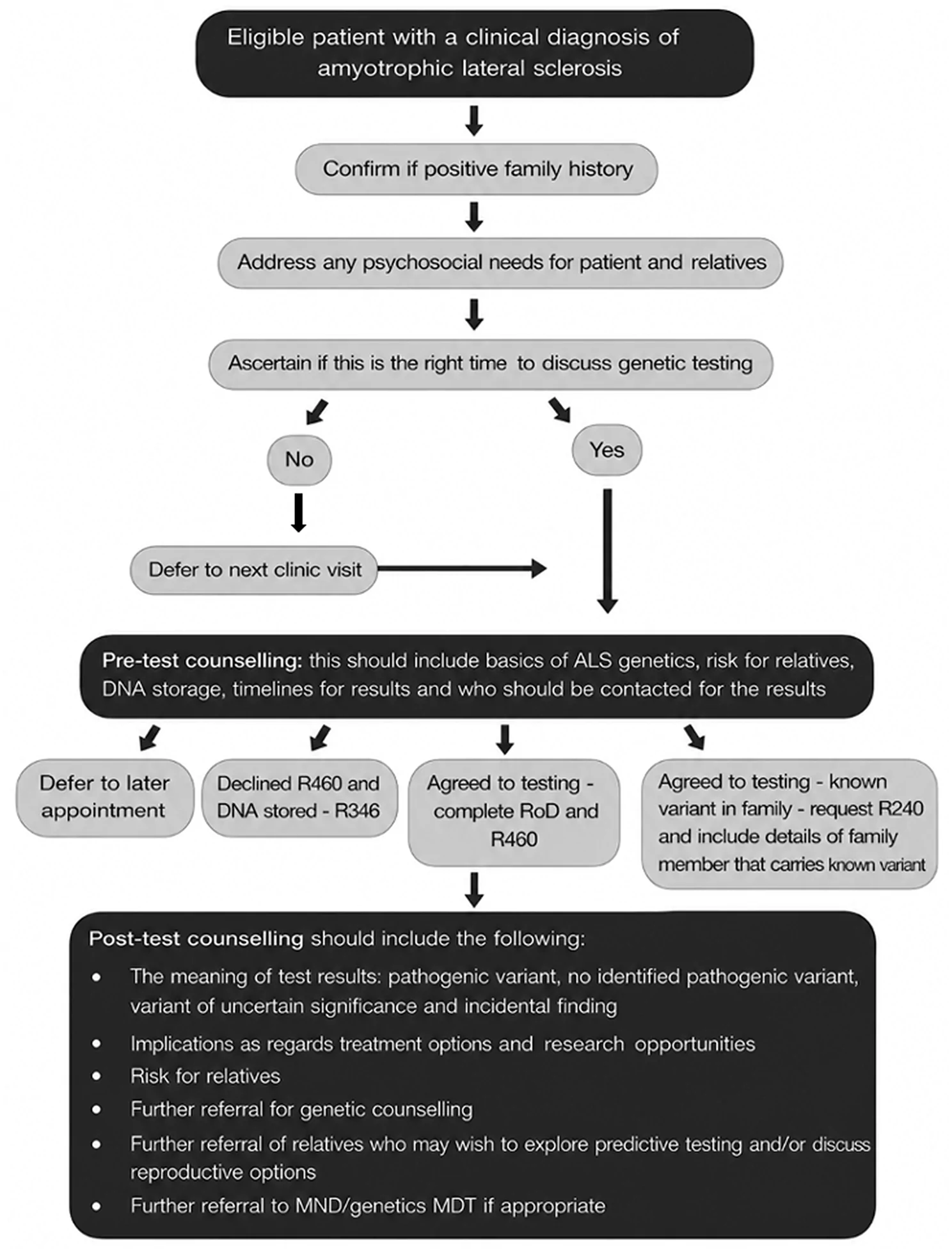
Flowchart outlines the optimised care pathway for genetic testing for individuals diagnosed with ALS using the National Genomic Test Directory (UK): R460 is the Amyotrophic Lateral Sclerosis test code, R346 requests DNA to be stored, R240 diagnostic testing for known mutation

This pathway (Fig. 2) embeds genetic testing within the MND clinic. Defined responsibilities are as follows:

- The neurologist identifies eligible patients, initiates discussions regarding genetic testing, completes consent documentation, and communicates their results.
- The MND nurse supports with pre-test discussions, facilitates communication among team members and patients, and coordinates referrals as needed.
- The genetic counsellor provides in-depth counselling to families when a pathogenic or likely pathogenic variant is identified.
- The Genomic Laboratory Hub team manages genetic testing procedures, reporting of results, and addresses technical queries.
- The joint MND/genetics multidisciplinary team (MDT) reviews complex cases, addresses ethical considerations, and evaluates uncertain results.

Implementation of the care pathway will be supported through a structured rollout within the MND clinic. This will include the following:

- Targeted training sessions for neurologists and MND allied healthcare professionals focusing on pre-test discussions, consent processes, and communication of results.
- Documentation templates and checklists will be embedded within electronic patient records to promote standardisation in recording genetic discussions and outcomes.
- DNA storage will be offered to all patients in their first visit as a default.
- Clear referral pathways to clinical genetics services will be established and referral for genetic counselling will be fast-tracked for ALS patients.
- Regular multidisciplinary team (MDT) meetings will be used to review complex cases and reinforce shared learning.

In the initial phase, pathway adoption will be piloted within the Manchester MND Care Centre, with ongoing feedback collected from clinicians and patients to inform refinement prior to wider implementation.

### Phase 4: Key performance indicator definition

A set of Key Performance Indicators (KPIs) was established to monitor implementation fidelity, assess the impact of the pathway, and ensure ongoing quality improvement. These KPIs are categorised into two domains and were derived from observations during the service evaluation and patient survey.

Process indicators assess the operational performance of the pathway and include the following:

- Uptake of genetic testing: the proportion of eligible persons who undergo testing after pre-test discussions.
- Uptake of DNA storage: the proportion of people who choose DNA banking instead of immediate testing.
- Turnaround time: the average interval between test referral and communication of the results to the patient.

Outcome indicators measure the experiential and qualitative impact of the pathway, including the following:

- Patient-reported experience measures: feedback collected through structured surveys.
- Staff-reported experience measures: assessment of staff confidence, perceived workload, and adequacy of training to deliver genomic testing within MND clinics.

KPIs will be monitored prospectively following implementation of the care pathway. We plan to carry out annual evaluations to assess sustainability and longer-term impact. Data collection will include repeat patient and staff surveys, adapted from the initial service evaluation to enable direct comparison of experiences before and after pathway introduction. In addition, retrospective chart reviews will be done to assess documentation completeness, uptake of genetic testing, and turnaround times.

## Discussion

### Summary of current practice

Genetic testing is increasingly recognised as an important component of care for individuals diagnosed with ALS, particularly as understanding of the genetic factors underlying the disease expands [2]. The service evaluation demonstrates meaningful uptake of genetic testing. Integration of the National Genomic Test Directory has standardised access to testing; however, variability persists in the content, timing, and documentation of the testing process.

### Identified gaps

Findings from the service evaluation and patient engagement session reveal inconsistencies and a lack of closure in genetic discussions, particularly following uninformative results. The emotional burden at diagnosis and time constraints in clinics often hinder comprehensive engagement with genomic testing, reflecting previously reported gaps across the UK.^6^ The rapid pace of genetic discoveries and emerging therapeutic opportunities further emphasises the need for timely and clear communication with individuals living with ALS and their relatives, many of whom may consider predictive or reproductive options. A recent survey confirmed that many neurologists lack formal training in genomics and report low confidence in conducting genetic testing conversations or in managing the complex consent process [11]. These findings highlight the need for specific tools and training to support clinicians in navigating these discussions. Additionally, processes for recontacting test recipients and their families as variant classifications change remain underdeveloped, presenting challenges for longitudinal care [12].

### Addressing the identified gaps

Development of the pathway was informed by findings from the service evaluation and patient survey. Gaps in patient recall, particularly regarding familial implications, result uncertainty, and next steps, led to the inclusion of structured pre- and post-test discussions in the pathway, supported by a standardised documentation checklist to prompt clinicians. Patient-reported uncertainty around timelines and testing logistics is addressed through explicit communication of expected turnaround times and a clear plan for result delivery at the point of consent.

An additional challenge in ALS genetic counselling is the reduced penetrance and pleiotropy associated with several ALS-related genes, including repeat expansions in *C9orf72 *[2]. This variability introduces uncertainty when discussing risk with patients and their relatives. The pathway addresses this by emphasising transparent communication of uncertainty during pre-test discussions and acknowledging the limitations of current risk prediction. For complex cases or confirmed pathogenic variants, referral to specialised clinical genetics services is recommended.

The care pathway offers an organised and trackable process that enables people living with ALS and their families to determine their current stage in the genetic testing process [13]. To accommodate future developments, the pathway facilitates regular reanalysis of stored DNA and ongoing recontact, ensuring that individuals and their families benefit from emerging genetic knowledge [14].

### Predictive testing

When a pathogenic or likely pathogenic variant is identified in a patient, predictive testing becomes available for adult relatives. This testing is accessed through Clinical Genetics services, with families supported by specialist Genetic Counsellors throughout the process. Predictive testing is typically discussed over several appointments, covering topics such as the psychosocial implications of genetic testing, available reproductive options, and different strategies for communicating genetic information to children. Although there are currently no evidence-based guidelines for predictive testing in ALS, Clinical Genetics services possess experience in advising families affected by various neurogenetic conditions with no cure or limited treatment options [15]. Further longitudinal research is required to understand how individuals and their families experience predictive testing. The literature has called for the development of predictive testing guidelines specific to ALS [16]. The extent to which general guidelines can be adapted, with certain elements tailored to the specific condition, requires further investigation. It is important to recognise that while guidelines are valuable, they are not prescriptive and require expert clinical judgment for appropriate application.

### Training and resources

Since completion of the service evaluation in 2024, several practical measures have been implemented to support delivery of the pathway and training of clinicians:

- **Targeted training**: genetic testing training (including the consent process) has been delivered to MND nurses to support them during consultations.
- **Multidisciplinary collaboration**: a regular, joint MND/clinical genetics MDT meeting has been established to discuss complex cases and facilitate shared learning between specialties.
- **Patient and clinician resources**: educational webinars, patient information leaflets, and an animation have been developed in collaboration with the MND Association and are now routinely used in clinic to support genetic testing discussions.
- **Rapid access to genetic counselling**: referrals for MND patients are now fast-tracked, with remote consultations offered where appropriate to reduce travel burden and enable participation of family members.

### Collaboration with patients and their families

The pathway is founded on a close partnership between clinical teams, individuals living with ALS, and their families. Ongoing patient involvement will be supported through follow-up surveys and patient workshops conducted on an annual basis, based on our defined KPIs.

## Limitations

The respondent cohort appears broadly representative of a typical ALS clinic population in terms of age distribution, with the majority aged between 60 and 80 years, and a balanced gender distribution. Most respondents had been living with the disease for more than one year, which may have influenced recall of earlier genetic discussions and contributed to the variability in reported experiences. Additionally, the low proportion of respondents with a known family history of ALS is consistent with the predominance of sporadic disease but may limit insights into the experiences of individuals from familial ALS backgrounds.

However, the study has some limitations that should be considered when interpreting the findings. The patient survey had a relatively low response rate (28/80, 35%), which may limit the generalisability of the results. As participation was voluntary, responses may be subject to selection bias, with individuals who had particularly positive or negative experiences more likely to respond. The survey also relied on self-reported recall, subjecting it to recall bias, particularly given the context of a complex and emotionally challenging diagnosis such as ALS. This may partly explain discrepancies between documented clinical practice and patient-reported understanding.

Also, findings from the service evaluation were solely captured from available documented conversations within clinic letters, and does not consider conversations that may have taken place outside of clinic.

In spite of these limitations, the consistency of themes identified supports the relevance of these results to pathway development.

## Conclusions

Implementation of an ALS genetic testing care pathway has the potential to standardise and improve the equity of genomic care for people living with ALS. Sustained success will require ongoing attention to staff training and continued collaboration across neurology, genetics, and patient communities. Subsequent evaluation of the pathway should be guided by clearly defined success criteria, which can be categorised into patient-reported, and staff reported measures. Patient-reported outcomes, namely improved understanding of genetic testing, clearer expectations regarding next steps, and greater satisfaction with communication, will be assessed as indicators of pathway effectiveness. In parallel, staff-reported measures, including increased confidence in delivering genetic counselling components within routine clinics will provide important insight into implementation success.

Future studies should assess the psychological, ethical, and service-level impacts of mainstreamed genetic testing, including predictive testing for at-risk relatives. Structured pathways of this nature may offer a scalable model for integrating genomics into the care of rare diseases across various healthcare systems.

## Data Availability

The data available is presented in the manuscript.

## Abbreviations

ALS: Amyotrophic Lateral Sclerosis
MND: Motor Neuron Disease
ROD: Record of Discussion
KPI: Key Performance Indicator
MDT: Multidisciplinary Team
CHCHD10: Coiled-coil-helix-coiled-coil-helix domain containing 10
TARDBP: TAR DNA-binding protein 43
SOD1: Superoxide Dismutase 1
UK: United Kingdom
RaERN: Rare European Reference Networks

## References

[1] 1, Brown RH, Al-Chalabi A. Amyotrophic lateral sclerosis. N Engl J Med. 2017;377(2):162–72. 10.1056/nejmra1603471. 28700839

[2] Chaouch A, Crook A, Andrew, McDermott CJ, Ammar Al-Chalabi, McNeill A, et al. The use of genetic testing in amyotrophic lateral sclerosis (ALS): a practical approach. Amyotroph Lateral Scler Frontotemporal Degeneration. 2025;1–7. 10.1080/21678421.2025.2539895 40779613

[3] Akçimen F, et al. Amyotrophic lateral sclerosis: translating genetic discoveries into therapies. Nat reviews Genet vol. 2023;24:642–58. 10.1038/s41576-023-00592-y. PMC10611979 37024676

[4] Alder J, Chukwuma C, Farragher T, Holden Smith S, Morris R, Ealing J, et al. Impact of relative deprivation and ethnicity on the incidence rate of amyotrophic lateral sclerosis. Amyotroph Lateral Scler Frontotemporal Degeneration. 2025;26(5–6):558–65. 10.1080/21678421.2025.2465609 39969486

[5] NHS England. National Genomic Test Directory [Internet]. England.nhs.uk. 2018. Available from: https://www.england.nhs.uk/publication/national-genomic-test-directories/

[6] De Oliveira, Hugo M, et al. A survey of current practice in genetic testing in amyotrophic lateral sclerosis in the UK and Republic of Ireland: implications for future planning. Amyotroph lateral Scler frontotemporal degeneration vol. 2023;24:5–6. 10.1080/21678421.2022.2150556. 36458618

[7] Optimal clinical pathway for adults: Neurogenetics National Neurosciences Advisory Group (NNAG) [Internet]. 2023 [cited 2026 Jan 5]. Available from:

[8] Talarico R, Cannizzo S, Lorenzoni V, et al. RarERN Path: a methodology towards the optimisation of patients’ care pathways in rare and complex diseases developed within the European Reference Networks. Orphanet J Rare Dis. 2020;15(1):347. 10.1186/s13023-020-01631-1. Published 2020 Dec 14. 33317578 PMC7734838

[9] Record of Discussion Regarding Genomic Testing [Internet]. Available from: https://www.england.nhs.uk/wp-content/uploads/2021/09/nhs-genomic-medicine-service-record-of-discussion-form.pdf

[10] Developing a patient decision aid to support genetic testing in MND [Internet]. MND Association. 2022 [cited 2025 Dec 26]. Available from: https://www.mndassociation.org/research/our-research/research-we-fund/improving-standards-of-care/decision-aid-for-genetic-testing

[11] Howard J, Bekker HL, McDermott CJ, McNeill A. Survey of service needs to embed genome sequencing for motor neuron disease in neurology in the English National Health Service. J Med Genet. 2024;61(7):661–5. 10.1136/jmg-2023-109735. Published 2024 Jun 20. 38458755 PMC11228195

[12] Bombard Y, Brothers KB, Fitzgerald-Butt S, et al. The Responsibility to Recontact Research Participants after Reinterpretation of Genetic and Genomic Research Results. Am J Hum Genet. 2019;104(4):578–95. 10.1016/j.ajhg.2019.02.025. 30951675 PMC6451731

[13] Choukair D, Hauck F, Bettendorf M, et al. An Integrated clinical pathway for diagnosis, treatment and care of rare diseases: model, operating procedures, and results of the project TRANSLATE-NAMSE funded by the German Federal Joint Committee. Orphanet J Rare Dis. 2021;16(1):474. 10.1186/s13023-021-02092-w. Published 2021 Nov 12. 34772435 PMC8588640

[14] Daoud H, Valdmanis PN, Gros-Louis F, et al. Resequencing of 29 candidate genes in patients with familial and sporadic amyotrophic lateral sclerosis. Arch Neurol. 2011;68(5):587–93. 10.1001/archneurol.2010.351. 21220648

[15] MacLeod R, Tibben A, Frontali M, Evers-Kiebooms G, Jones A, Martinez-Descales A, et al. Recommendations for the predictive genetic test in Huntington’s disease. Clin Genet [Internet]. 2013;83(3):221–31. 22642570 10.1111/j.1399-0004.2012.01900.x

[16] McNeill A, Amador MM, Bekker H, Clarke A, Crook A, Cummings C et al. Predictive genetic testing for Motor neuron disease: time for a guideline? European Journal of Human Genetics [Internet]. 2022;30(6):635–6. Available from: https://www.nature.com/articles/s41431-022-01093-y 10.1038/s41431-022-01093-y PMC9177585 35379930

